# Distances to emergency departments and non-urgent utilization of medical services: a systematic review

**DOI:** 10.1080/16549716.2024.2353994

**Published:** 2024-06-03

**Authors:** Uma Kelekar, Debasree Das Gupta, Nicole Theis-Mahon, Emily Fashingbauer, Boyen Huang

**Affiliations:** a Innovation, Leadership and Technology/Center for Optimal Aging, Marymount University College of Business, Arlington, VA, USA; b Kinesiology and Health Science Department, Utah State University Emma Eccles Jones College of Education and Human Services, Logan, UT, USA; c Health Sciences Library, University of Minnesota, Minneapolis, MN, USA; d Minnesota State University College of Allied Health and Nursing, Mankato, MN, USA; e University of Minnesota School of Dentistry, Minneapolis, MN, USA

**Keywords:** Distance, distance decay effect, emergency department, non-urgent utilization, systematic review

## Abstract

**Background:**

The use of Emergency Departments (EDs) for non-urgent medical conditions is a global public health concern.

**Objectives:**

A systematic review, guided by a registered protocol (PROSPERO: CRD42023398674), was conducted to interpret the association between distance as a measure of healthcare access and the utilization of EDs for non-urgent care in high- and middle-income countries.

**Methods:**

The search was conducted on 22 August 2023 across five databases using controlled vocabulary and natural language keywords. Eligibility criteria included studies that examined non-urgent care, and featured concepts of emergency departments, non-urgent health services and distance, reported in English. Articles and abstracts where patients were transported by ambulance/paramedic services, referred/transferred from another hospital to an ED, or those that measured distance to an ED from another health facility were excluded. The Grading of Recommendations, Assessment, Development, and Evaluations (GRADE) framework informed the quality of evidence.

**Results:**

Fifteen articles met the inclusion criteria. All studies demonstrated satisfactory quality with regard to study design, conduct, analysis and presentation of results. Eight (53.3%) of the studies (1 paediatric, 4 all ages/adult, 3 ecological) found a moderate level of evidence of an inverse association between distance and ED visit volume or utilization for non-urgent medical conditions, while the remaining studies reported very low or low evidence.

**Conclusions:**

Half of the studies reported non-urgent ED use to be associated with shortest distance traveled or transportation time. This finding bears implications for healthcare policies aiming to reduce ED use for non-urgent care.

## Paper context

- **Main findings:** Using the systematic review methodology, a qualitative synthesis of the prior literature showed moderate evidence of the negative role of distance on emergency departments use across middle- and high-income countries.
- **Added knowledge:** This systematic review adds to the literature by providing evidence that clarifies prior inconsistent findings on the association between distance, a measure of healthcare access, and non-urgent emergency department utilization.
- **Global health impact for policy and action:** Based on the main finding of negative role of distance, a three-tiered policy recommendation to reduce non-urgent use of emergency departments is provided that include: 1) public health systems reforms, 2) patient education, and 3) improved access to primary care providers.

## Background

Emergency departments (ED), alternatively referred to as emergency rooms (ER), hospital emergency services, and accident and emergency departments (A&E), are primarily designed to treat urgent, traumatic, or life-threatening medical conditions [1]. However, ED visits for non-urgent medical conditions, termed as ‘inappropriate ED use’, is increasing and becoming a public health concern [1]. Non-urgent ED visits are typically defined as visits for conditions for which a delay of several hours would not increase the likelihood of an adverse outcome [2]. In countries such as the United States or France, lack of universal or comprehensive healthcare coverage is associated with a higher non-urgent utilization of EDs by some segments of the population, such as minority groups [3], low-literacy adults [4], individuals of low socio-economic status [5], and the uninsured [6]. Lack of access to primary care providers [7], inadequate understanding of medical urgency [8], or lack of access to any care and subsequent reliance on EDs for basic medical needs [5] are additional factors that contribute to non-urgent use of EDs in high and middle-income countries with universal health coverage [5,7].

Prior studies have also shown that the distance to EDs, interpreted as ‘access’ to health services, may influence one’s decision to use EDs for non-urgent care [9,10]. Theoretical support underpinning these empirical findings appear in Andersen and Newman’s model of Health Services Use [11] in which ‘access’ to health providers and facilities is an ‘enabling factor’ that determines utilization of health services, such as ED care. A corollary of this theory indicates that access to and utilization of health services will decline with increasing distance to health facilities [12,13]. The adverse role of distance in lowering access to and utilization of health services is related to travel time and transportation costs that individuals incur when traveling from their residence or workplace to a healthcare facility, such as an ED, to obtain care. Longer travel time and higher transportation costs impose greater ‘travel costs’, which create a ‘tax-like’ effect to lower demand for health services [12].

The ‘tax-like’ deterrent effect of travel distance may be especially true if the medical reason for the ED visit is of non-urgent nature [12]. This inverse relationship between travel distance and healthcare utilization is termed in the literature as the ‘*distance decay effect*’ [7,12,13]. The term ‘*distance decay effect*’ was most likely used for the first time by Stock to describe the influence of travel distance on the utilization of health care facilities [14]. The effect of distance decay was considered particularly impactful in rural areas of developing countries, where the supply of healthcare services was extremely sparse [14]. As EDs are outnumbered by primary care facilities and hospitals in every developed country, their functional roles are too unique to be replaced by primary care facilities and hospitals. Thus, the relative sparseness of ED facilities may explain why ED utilization is prone to a distance decay effect, especially for non-urgent services.

Understanding the role of distance in influencing non-urgent use of ED services is vital for several reasons. First, prior studies report inconsistent findings, partly due to the fact that distance decay phenomenon has been studied in the context of single states within a country or single sites of care, such as a hospital system [13,15]. Second, the nature of the distance decay phenomenon may change depending upon the geographic size of the country studied. For instance, in larger geographical countries such as the United States, distances traveled between any two locations tend to be longer compared to countries that are smaller in geographic size [16]. Hence, examining evidence on distance decay effects across multiple countries is valuable for a comprehensive and robust picture on the role of distance in ED use for non-urgent care. Furthermore, the use of EDs for non-urgent reasons may have several undesirable consequences such as overcrowding [17], inefficient use of resources [18], poor health outcomes [19], and high spending [20]. Providing care in an ED setting is costlier than in outpatient settings such as primary care clinics in the community [20,21]. Thus, understanding whether distance is a factor in influencing non-urgent use of EDs among vulnerable or disadvantaged populations without access to primary medical care is paramount. Finally, this insight could inform policy interventions aiming to reduce non-urgent use of EDs directly through patient education [22] or indirectly via location-allocation planning of future EDs without negatively affecting the overall health of the population served [23].

While a systematic evaluation to summarize findings from prior works could provide clarity on the role of distance in ED utilization for non-urgent health services, previous systematic reviews on utilization of ED services have focused on different socio-demographic and clinical factors associated with the use of non-urgent ED care [24,25]. Consequently, the role played by distance in ED use for non-urgent care remains less than clear. To address this gap, we focus on the role of distance as an important enabling factor in determining non-urgent ED use. Guided by Andersen and Newman’s Model of Health Services Use, we apply the systematic review methodology to narratively synthesize prior findings on the role of distance in non-urgent use of EDs across different sub-groups of populations and multiple countries.

## Methods

### Search strategy

The protocol of this systematic review was registered in PROSPERO (CRD42023398674), an international prospective of systematic reviews [26] and followed the Preferred Reporting Items for Systematic Reviews and Meta-analyses (PRISMA) checklist methodology to report findings [27]. We conducted a literature search using a combination of controlled vocabulary and natural language in accordance with the Cochrane Handbook for Systematic Reviews of Interventions [28]. The search strategy was developed by a librarian (NTM) and included search terms covering the concepts of emergency departments, non-urgent health services, and distance (See Figure 2). This search strategy was developed using Medline via the Ovid interface (Ovid Medline® ALL), and then translated across five databases that included: Embase via Ovid (Classic+Embase), CINAHL, Scopus, Web of Science (Core Collection), and Cochrane CENTRAL. The search window for this systemic review spanned the entire publication period between individual database inception and our search date (22 August 2023). The Medline via Ovid search strategy is available in Table 1. The search results were exported to Covidence for deduplication and screening [29].

**Table 1.** Ovid Medline® ALL search strategy.

Concept	MeSH (controlled vocabulary)	Natural language keywords
Emergency department	exp Emergency Service, Hospital/or exp Emergency Medical Services/or exp After-Hours Care/	(emergency adj4 (department* or room* or ward* or unit* or service*)) or ((health or medical) adj5 emergenc*) or ER
Non-Urgent use	exp health services misuse/	(non-urgent or nonurgent or non-traumatic or nontraumatic or non-emergency or nonemergency) or (avoidable or inappropriate)
Distance	exp Travel/	(travel or distance or transport*)

Table Key.

*truncation of word.

adj is adjacency search (e.g. adj4 means within four words, adj5 within 5 words)

Records were screened independently by two reviews (UK and BH) and conflicts were resolved through consensus. After title and abstract screening, full text for the remaining articles was retrieved. Two reviewers, UK and BH, assessed the remaining articles against study inclusion criteria. Non-English, conference abstracts, articles where all patients were transported by ambulance or paramedic services, referred or transferred from another hospital to an ED, or those that measured distance to an ED from another health facility were excluded. Inclusion criteria included studies (i) investigating non-urgent, non-traumatic, inappropriate, or avoidable utilization of ED services, and (ii) measuring distance from a patient’s residential address or zip code to an ED facility.

### Conceptual framework

**Figure 1.**
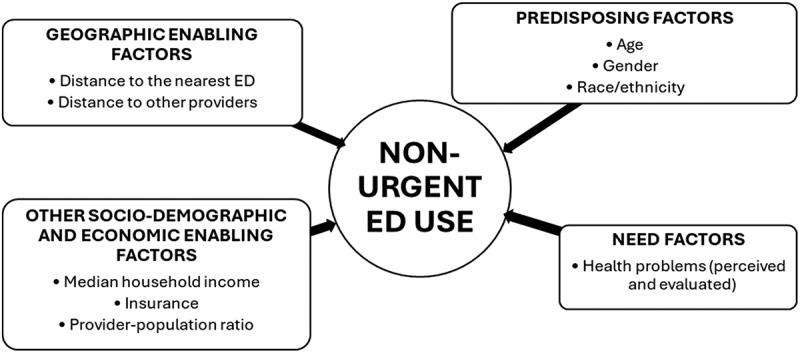
Proposed conceptual framework adapted from the Andersen and Newman’s Model of health services use.

This systematic review was guided by Andersen and Newman’s Model of Health Services Use [11] (Figure 1), a comprehensive framework defining health utilization behavior based on individual and environmental contextual characteristics classified into three categories: predisposing, enabling and need factors that collectively influence individuals’ behaviors encompassing the use of health services. As opposed to individual characteristics, environmental contextual characteristics are aggregate or ecological level community factors. Predisposing factors, such as age, sex, race/ethnicity, are individual socio-demographic characteristics and community compositions that exist prior to an illness to predispose health service use among individuals and in communities. Enabling factors include individual (such as insurance) and contextual (such as, provider–population ratio, and median household income) economic characteristics that facilitate use of health services. Enabling factors also include healthcare access characteristics, such as distance to healthcare providers and facilities including EDs. Need factors, such as perceived health status, are the most immediate causes of healthcare use resulting from perceived or evaluated health problems. Grounded in this widely used framework to study utilization of ED health services, this systematic review will narratively synthesize prior findings on the role of travel distance as a determinant of ED use for non-urgent care.

### Data extraction

Data extracted by UK and BH from the selected articles included: (i) definition for non-urgent ED visits and distance (Table 2), (ii) study characteristics (study authors, country where the study was conducted, type of research design, study aim, study period, and study sample), (iii) data collection methodology (iv) key findings on non-urgent ED utilization rate, average distance traveled to an ED and distance decay effect, and (v) potential confounders. Additionally, key findings were classified to report on effect of distance decay for (i) patient-level for pediatric populations, (ii) patient-level for adult/all ages populations, and (iii) ecological or aggregate-level for larger spatial units, such as census tracts or patient zip-codes. For studies that used an ecological study design, data on populations or groups was analyzed rather than individual data. Due to the heterogeneous nature of the studies included, conducting a meta-analysis was not feasible.

**Table 2.** Key findings from the reviewed studies.

Author/Country, Year	Design/Patient or Ecological level	Aim of the study	Study period (Months/Year)	Sample description	Final number of participants	Outcome variable	Definition of non-urgent	Distance Measurement	Key findings	Potential Confounders in the multivariable analysis^^
Valent & Busolin, Italy, 2018 [9]	Cross sectional/Pediatric ecological study	To assess how distance from home to the closest PED affects non-urgent visits among children.	2014	Children from 3 regional pediatric EDs <16 years with a white triage tag and residing within 20 kms^ of the ED	10,327	Non-urgent ED utilization rate	White triage tags	Patient home to ED	1)The proportion of ED non-urgent visits accounted for 24.7% of all visits. 2) The rate of non-urgent visits decreased significantly from the innermost distance band (163.5 ED visits/1,000 person-years) to outermost distance band. (26.6 ED visits per 1000 person years).	None
Hilker, US, 1978 [30]	Cross sectional/Pediatric patient-level study	Examine factors to explain why parents take their children to hospital EDs.	One week each during January and February, 1977.	Randomly selected parents of children visiting a single children’s hospital during two weeks in a 1-year duration	652 parents, and children	Travel time	Patient to whom a delay of 24 hours would make no appreciable difference in the clinical condition.	Travel time	1) Of the non-emergency visits, 61% of the children took less than 15 minutes to get to the ED.	None
Pehlivanturk-Kizilkan et al. Turkey, 2022 [31]	Cross sectional/Pediatric patient-level study	To evaluate the factors that might have an effect on non-urgent PED visits and parental overestimation of emergency severity.	January-June 2015	Children patients who visited a district state hospital during the shifts of 2 physicians (who also assessed the emergency status of all patients over a 6-month duration)	974 patients	Non-urgent ED visits	Pediatric Canadian Triage and Acuity Scale (PCTAS)	ED to patient home	68% of the ED visits were classified as non-urgent. 2) 92.6% of the children lived close to the ED i.e. inside the district border. 3) Distance decay effect was not significant.	**Age**, Gender, **Presence of chronic disease**, Time of admission Season of admission, **Parents’ age**, Parents’ literacy, **Father’s working status** **Admission to ED in past week**, Total number of children, Transportation method
Benahmed et al. Belgium, 2012 [32]	Cross sectional/ Pediatric patient-level study	To evaluate the magnitude of non-urgent use of the ED for pediatric patients and identify associated factors.	2 weeks in October and November 2010	All children <15 years of age were randomly selected across 12 Belgian hospitals	3,117	Non-urgent utilization rate	ED attendance was appropriate when at least, one of the following criteria was met: need for a short-stay observation, need for technical examination (X-rays, blood testing, etc.) or orthopedic/surgical treatment, in-patient admission, death, referral by doctor or police, or brought by ambulance.	Travel time from patient home to ED	1) The non-urgent utilization was 39.9%. 2) 87.6% of those visiting the ED for non-urgent reasons took a travel time of less than 30 minutes. 3)The effect of distance on ED use was negative and significant. 4) Those whose travel time was less than 30 minutes, had higher odds (OR = 1.7 [95% CI = 1.3–2.2) of visiting the ED than those with travel times of 30 minutes or more.	**Age**, Disadvantaged family **Hospital setting** **Out-of-hours visit Registered family doctor**
Guckert et al. Germany, 2022 [33]	Cohort study/ Pediatric patient-level study	To determine factors leading to a non-urgent presentation at the ED before and during the first peak of the COVID-19 pandemic.	January-December 2017; March-April 2020	Children 16 years or less visiting a single university hospital and residing within a geographic distance between 0–20 kms.	5038 unscheduled visits	Non-urgent ED visits	Manchester Triage System Algorithm (MTS)	Pediatric ED to the patient’s zip code area’s geographical center	1) 69% of non-urgent visits among pediatric population 2) Non-urgent ED use decreased by 68% from pre-COVID to first peak of the COVID-pandemic 3) 50% of the patients traveled less than 5 kms for non-urgent visits. 4) For children with non-urgent presentations, median distance to the pediatric ED was 5.4 km 5) The distance decay effect was negative and significant.	**Age**, Sex, **Absence of pre-existing conditions**, **ED visit during PCP office hours.**
Wartman et al. US, 1984 [34]	Other: Mixed methods: cross-sectional and interviews/All ages patient level study	To characterize the leavers by examining in detail socioeconomic factors, medical problems, and distance traveled.	One year period	All patients presenting to a university affiliated hospital ED.	335 matched pairs of leavers and stayers/Interviews with 100 leavers	Leaver is identified as one who leaves without treatment.	Non-urgent complaints were graded by level (1–6) according to a classification developed by the ED based on methods and time needed for treatment.	ED to patient home	**1)** Leavers lived within 2.5 miles of the hospital. 2) 68% percent lived in the first five concentric zones (or within 2 ½ miles of the hospital) compared to 53% of stayers. 3) Half of the leavers living within 2.5 miles of the hospital presented with a mild illness. 4) Multiple discriminant analysis found distance to be one of the distinguishing variables between leavers and stayers.	None
Bianco et al. Italy, 2003 [35]	Cross sectional, Adult patient-level study	Determine the extent of non-urgent visits and effect of different characteristics on such visits.	July 2001-December 2001	Patients (≥15 years) waiting for care in an ED of a 714-bed hospital. Stratified random sample was selected to include 2-h sessions during two weekdays and one weekend for each week of the study period.	500 patients, relatives provided information for 41 patients.	Non-urgent utilization rate	A non-urgent case was defined as one where the patient has no active symptoms or they were recent and minor, without any feeling of emergency and he/she desires a check-up, a prescription refill or a return-to-work release.	ED to patient home	1) 19.6% of the people had non-urgent conditions. 2) The percent of non-urgent visitors declined with increasing distance bands. 3) For non-urgent visits, 23.1% traveled 5 kms or less versus 20% traveled over 35 kms. 4) In the multivariate analysis, the effect of distance on ED use was negative [OR = 0.88, 95% CI 0.65–1.18], but not significant.	**Age**, **Sex**, Chronic disease, Day of the week, Education, **Duration of presenting problem**, **Referral to the ED**, Number of persons in a household
Naouri et al. France, 2020 [5]	Cross sectional/Adult patient-level study	To explore socioeconomic and geographical determinants of inappropriate ED use in France.	All visits that took place on June 11 (Tuesday), 2013	All patients ≥15 years who visited a French ED during the 24-hour across 734 EDs	29,407 patients	Inappropriate ED use	Three measures of ED use appropriateness: Appropriate Use, Possible GP Use, Resource Utilization.	ED to patient home	**1)** Based on three methods, 23.6%, 27.4%, 13.5% were considered to be inappropriate ED use, and 6.16% to be inappropriate according to all three measures. **2) 63–66% who visited the ED for inappropriate use traveled 10 kms or less**. **3)** Distance decay effect was negative and significant. **4)** When compared to those living 10 kms or less, those living more than 10 kms away, were less likely to use the ED inappropriately on the three measures of appropriateness: [OR = 0.89***, 95% CI 0.82–0.95]; [OR = 0.85***, 95% CI 0.79–0.91]; [OR = 0.83***, 95% CI 0.76–0.91];	**Sex**, **Age**, Insurance type, **Having supplemental insurance** **Having a GP, Employment status**, **Education**, **Place of residence**, **Onset of complaint before the day of the ED visit**, **Time of visit**, **Type of hospital**, **Annual number of ED visits**, County medical density.
Oh et al. Korea, 2018 [36]	Cohort/ Adult patient level study	Analyze the demographic, cancer-related and clinical characteristics associated with cancer patients visiting the ED.	January-December, 2016	Adult patients ≥19 years diagnosed of cancer visiting a tertiary care hospital	4,346 patients	Avoidable ED visit	A visit to the ED with a problem that could be resolved at a primary care office, out-patient clinic, or over the telephone.	ED to patient home	**1)** About 55.7% of the visits were avoidable, and 90.5% of the visits were primary-care treatable. **2)** Approximately 38.9 kms were traveled for avoidable visits. **3)** The effect of distance on ED use was negative and significant. **4)** With a 1-km longer distance to home from ED, the probability of avoidable ED visits dropped by 0.2% (odds ratio [OR] 0.998, 95% CI: 0.997–0.999]	Gender, BMI **Type and status of cancer**, **Chief complaint**, **ED visit in the last one year**, **Stay in the ED**, **Marital status**, Final education level, **Laboratory and imaging tests in ED.**
Chen et al. US, 2015 (South Carolina) [10]	Longitudinal/All ages patient level study	To investigate whether convenience (proxied by travel distance to the hospital ED and to the closest Federally Qualified Health Center (FQHC) is associated with non-urgent ED use.	2006–2010	ED visits in South Carolina between 2005–2010.	6,592,501 ED visits	Probability associated with a NEPCT (Non-urgent or Primary Care Treatable) visit.	New York University – Emergency Department Algorithm (NYU-EDA)	Estimated shortest road distance between patient’s address and ED facility/FQHC visited.	**1)** The average distance traveled to the hospital ED was 11.685 miles, while that to a FQHC was 10.610 miles. **2)** As distance increased, the probability of using the ED for NEPCT visit declined. 3) When compared across different insurance types, the slope coefficients were highest for commercial insurance (β = ‒0.0432, SE = 0.007), followed by self-pay (β = ‒0.0699, SE = 0.007), and those with other payment (β = ‒0.103, SE = 0.02).	**Gender**, **Age**, **Race/Ethnicity, Insurance type**, **FQHC location** Day of the week^~^
Chen et al. US, 2015 (California) [37]	Longitudinal, All ages patient-level study	To examine if travel distances to the ED are associated with avoidable ED utilization and whether Medicaid patients’ avoidable ED utilization patterns differ when a FQHC exists within half a mile of patients’ zip code population centroid.	2006–2010	~7.2% of all ED visits randomly selected in California.	3,912,676 ED visits	Probability associated with non-emergent (NE) visits	New York University – Emergency Department Algorithm (NYU-EDA)	Estimated distance between ED facility or nearest FQHC and the 2008 population centroid of patient’s zip code	**1)** The average distance traveled to the ED and FQHC was 5.2 miles, and 4.8 miles respectively **2)** 23.3% of the ED visits were classified as NE in nature. **4)** For Medicare and Medicaid patients, for those who had to travel a short distance to get to the ED, there was a 0.44 [SE = 0.24] and 0.45 [SE = 0.22] percent points change respectively associated with NE care. **5)** For Medicaid patients, if a FQHC was located within 0.5 miles of the population centroid of patient’s zip code, there was a reduction of 0.38% points [SE = 0.14] associated with NE care.	**Age** **Gender** **Race or Ethnicity, Insurance**, **FQHC location**, Year and hospital fixed effects^~^
Vaz et al. Portugal, 2014 [38]	Longitudinal, Adult ecological study	To test for the Accident & Emergency (A&E) distance decay effects on the demand for ED in Portugal, and other determinants of ED utilization in Portugal.	January-June 2011, January-June 2012	ED visits of adults ≥18 years from 3 NHS hospitals (basic, medical-surgical, polyvalent EDs)	229,528 visits and 1,490 frequesias	ED utilization rate	Manchester Triage classification	ED to frequesias of patient’s residential address	**1)** Distance had a negative and significant effect on ED use across the three kinds of EDs examined. **2)** For all low severity visits, across the three kinds of EDs, the Incidence Rate Ratios (IRR) were as: Basic ED: IRR= −1.29***; Med-Sur ED:IRR = −2.24***, Polyvalent ED:IRR = −1.27***.	**Mean Age**, **Male population** **Type of ED** **Availability of doctors, primary health care, modern health centers**, **Income**, **School dropout rate** **Unemployment rate, Homelessness**, **Over-crowding of homes** **Mortality rate**, **Live births**, **Psychiatric care** **Ophthalmological care**, **Severity or Triage** **Year**
Okunseri et al. US, 2016 [15]	Longitudinal/All ages ecological study	To examine the role of distance in the use of EDs for NTDCs (Non-traumatic dental conditions)	2001–2009	NTDCs visits to ED of the Medicaid population of Wisconsin enrolled in fee-for-service program or managed care organization	4,565,594 person years 80,742 ED visits.	ED visits per person-year	NTDC of first primary diagnosis	Medicaid enrollee’s location to the nearest ED	**1)** The ED visits per person-year was 17.7 visits per 1000 person years. **2)** The median distance to the nearest ED was 2.9 miles. **3)** As distance increased, the risk ratio (RR) associated with ED use declined indicative of a distance decay. **4)** Compared to enrollees living less than 0.5 miles from the nearest emergency department, those living 3 miles away [RR = 0.88, 95% CI = 0.96–0.84] or further had significantly lower rates of NTDC visits to ED.	Sex^~^, Age^~^, Race or Ethnicity, Location, Year, Dentist-Population Ratio, Urbanization category
Ingram et al. Canada, 1978 [39]	Cross sectional/All-ages ecological study	Examine the patient’s decision to visit the ED, and study the effect of distance on the pattern of ED utilization	1 May 1975–2 June 1975	A randomly selected sample of ED visits from Humber Memorial Hospital	105	Patient visits per 1,000 population	Patient and medical assessment	Estimated distance between ED to patient’s census tract center	**1)** The average distance traveled to the ED was 2.37 miles. **2)** Over 54% of those who assessed their problem to be non-urgent resided within 3 miles of the hospital **3)** The effect of distance on ED use for all visits was negative and significant (β=-0.477 SE = 0.2796].	None
McGarry et al. US, 2023 [40]	Cohort/longitudinal adult ecological study	To examine the relationship between distance to the nearest hospital and Assisted Living (AL) residents’ rate of ED use.	2018–2019	Treat and release ED visits among Medicare beneficiaries aged ≥55 years residing in AL communities spending at least 7 days in 2018 or 2019.	540,944 resident years	ED utilization rate per 1000 AL Resident years	NYU ED Algorithm	Estimated shortest distance between AL and nearest US hospital	The median distance from AL facility to the nearest hospital was 2.5 miles or 4 kms. **2)** The distance decay effect was negative and significant. **3)** Doubling the distance from nearest hospital was associated with 3% [95% CI: −4.1%, −1.9%] decrease in NE visits.	Beneficiary level controls^~^, Assisted living characteristics^~^ (e.g. bed size; length of stay) Year fixed effects~

***: Statistically significant at 5% level of significance; ^ kms represents kilometers, where 1 mile = 1.609 kms, ^^ Bold letters signify a known statistical significance of the confounder, ^~^ Statistical significance not known, ED: Emergency Department, PED: Pediatric Emergency Department, NEPCT: Non-Emergent or Primary Care Treatable visit.

### Quality of evidence and appraisal of studies

To assess quality of the evidence in the reviewed studies, we applied the Grading of Recommendations Assessment Development and Evaluation (GRADE) guidelines, following Balshem et al. [41]. The five categories used to assess quality in GRADE are risk of bias, imprecision, inconsistency, indirectness, and publication bias. All except one study included in this systematic review used retrospective observational data. Thus, based on this classification, while the reviewed articles start from a ‘low’ level of evidence following GRADE, we graded the level of evidence in each individual study upward based on (i) the reported distance decay effect size, and (ii) confounding variables included to adjust the size and/or directionality of the reported effect. The quality of evidence was downgraded based on ‘serious’ or ‘very serious’ problems on one or more of the GRADE criteria related to inconsistent definitions of non-urgent ED use and/or distance, study being limited to a single site of care or a small sample (≤100 participants), data collected over a short duration (<1 week), or study being ecological as opposed to a patient-level analysis. The quality of evidence based on GRADE is presented in Table 3.

**Table 3.** Quality of evidence and risk of bias classification of studies based on GRADE criteria.

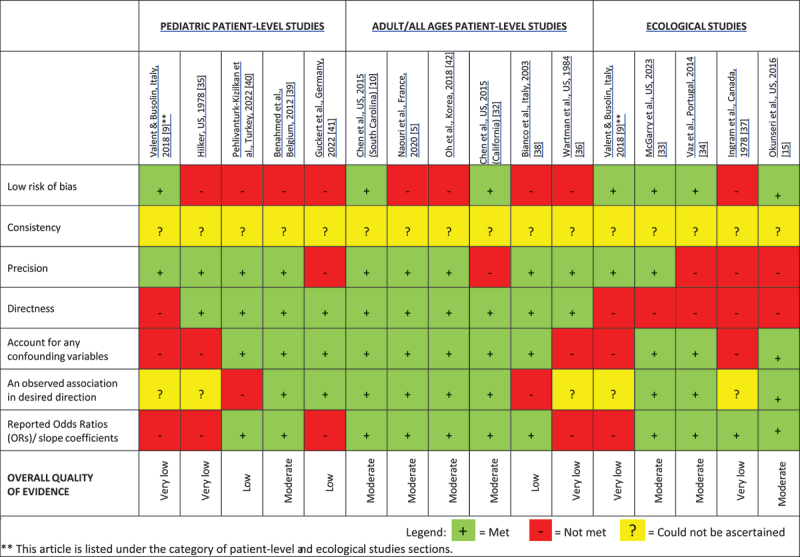

The quality of the included publications was evaluated by two reviewers (UK and BH) separately using an established quality appraisal tool [42], which comprised of fourteen criteria listed in Table 4. For each of the fourteen quality assessment criteria, a study was graded as ‘satisfactory’, ‘unsatisfactory’, or ‘unable to assess’. Each reviewer (UK and BH) entered their assessment into a Microsoft Excel spreadsheet and discrepancies were discussed and adjusted until consensus was reached.

**Table 4.** List of the assessment criteria and studies that were graded as satisfactory based on each assessment criterion.

Criterion	Satisfactory	(% of studies)
Clearly stated aims	[5, 9, 10, 15, 32-42]	100%
Appropriateness of design to meet the aims	[5, 9, 10, 15, 32-42]	100%
Adequate specification of subject group given	[5, 9, 10, 15, 32-42]	100%
Justification of sample size	[5, 9, 10, 15, 32-32]	100%
Likelihood of reliable and valid measurements	Unable to assess	Not applicable
Adequate description of statistical methods	[5, 9, 10, 15 ,32-42]	100%
Adequate description of data	[5, 9, 10, 32, 33, 35, 37-32]	80%
Consistency in number of subjects reported	[5, 9, 10, 32-40]	80%
Assessment of statistical significance	[5, 9, 10, 15, 32-40, 42]	93.3%
Attention to potential biases	None	0%
Meaningful main findings	[5, 9, 10, 15, 32-37, 39, 41, 42]	86%
Interpretations of null findings	[5, 9, 10, 15, 32-37, 39-42]	93.3%
Interpretation of important effects	[5, 9, 10, 15, 32-35, 37, 39-42]	86%
Comparison of results with previous reports	[5, 9, 10, 15, 32-34, 36-42]	93.3%
Implications in real life	[5, 9, 10, 15, 32-34, 36, 42]	93.3%

## Results

**Figure 2.**
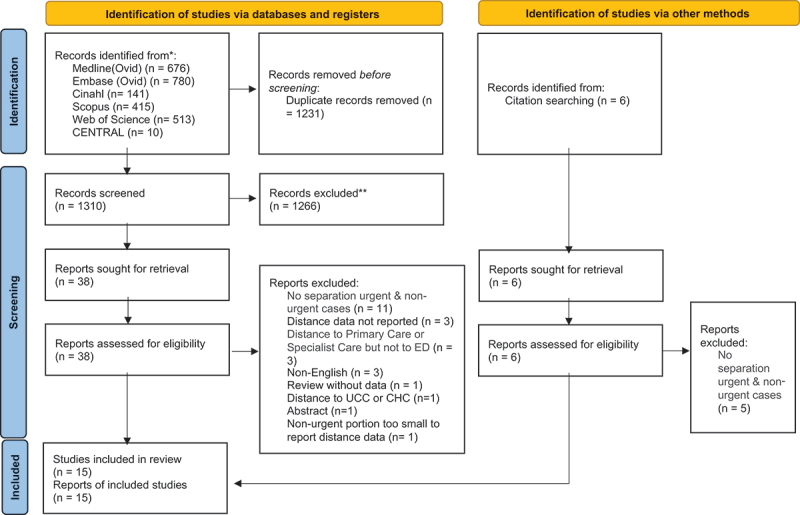
PRISMA flow chart of included studies.

The literature search identified 1310 articles after duplicates were removed. A total of fifteen studies were included in this review with additional details indicated in the PRISMA flow diagram (Figure 2).

### Study characteristics

Details on study characteristics are provided in Table 2. With the exception of five studies [10,15,37,38,40], all reviewed studies were cross-sectional in nature. Seven studies collected primary data [5,30–32,34,35,39] while eight studies relied on secondary data [9,10,15,33,36–38,40]. All studies were published between 1978 and 2023, and conducted in North America (USA [10,15,30,34,37,40] and Canada [39]), Europe (Italy [9,35], Belgium [32], Germany [33], Portugal [38], France [5], Turkey [31]), and Korea [36]. All the studies examined the role of distance on the utilization of non-urgent services in the ED.

### Definitions used to identify non-urgent ED visit and distance

A variety of methods were used to identify non-urgent visits across the fifteen studies. Six studies used an algorithm, such as the New York University – Emergency Department Algorithm (NYU-EDA) [10,37,40], the Manchester Triage System Algorithm [33,38], or the Pediatric Canadian Triage and Acuity Scale (PCTAS) [31], to define and select non-urgent ED visits. One study used primary diagnosis codes to define non-traumatic dental conditions seen in the ED [15]. The remaining eight studies defined inappropriate use by applying pre-determined criteria verified by a single or group of physicians [5,9,30,32,34,36,39], or applied criteria based on prior published studies [35]. Only two studies reported inter-rater agreement between physicians assessing non-urgency using the Cohen’s Kappa coefficient to be almost perfect [35,36]. Two studies reported the inter-rater agreement between physicians and patients (i.e. adults or parents of children) assessing the medical urgency of the reasons for visiting the ED [31,39]. While the study focusing on the pediatric population reported almost no agreement between parents of the children and physicians [31], another study that comprised of patients of all ages reported 36% of the complaints to be similarly assessed by physicians and the patients [39].

All studies reported a measure of estimated distance. Eight studies used distance traveled from patients’ residence to an ED [5,9,10,31,34–36] or Euclidean distance from a patient’s residence to the nearest ED [40]. The primary difference between Euclidean distance versus travel distance is that the former measures distance using the straight-line distance between two points, whereas travel distance measures distance using road networks as the travel distance between two points. Of all the studies, one reported measuring distance as ‘travel distance’ using Google maps to determine the shortest road distance traveled by patients [36], while others estimated distance as reported by the study participants in the surveys [5,31,34,35]. Additionally, two studies reported travel time based on the existing transportation networks, as reported by the study participants [30,32], while the remaining studies reported the average distance between the population centroid of a patient’s zip-code, municipality or census tract and the nearest ED facility [15,37] or the ED facility visited [33,38,39]. Based on where the study was conducted, distance was reported in miles (e.g. North America) or kilometers (e.g. Europe and Korea). The distance traveled to an ED or the nearest ED ranged between 2 and 24 miles (3.2–38.9 kilometers, 1 mile = 1.609 kilometers).

#### Data collection methods

Seven of the fifteen studies relied on observational retrospective data from a single hospital [30,31,33–36,39] while two studies collected data from patients visiting multiple hospitals or facilities [5,32]. Six studies utilized administrative ED databases or insurance claims databases comprised of data from multiple ED facilities [9,10,15,37,38,40]. Two studies combined socio-demographic patient-specific data with qualitative information retrieved through surveys or interviews such as motivations to visit an ED [5,34]. One study limited study participants to assisted living (AL) facilities residents and Medicare beneficiaries in the US [40]. Data collection time frames varied widely, ranging from multiple-years to a single day of a calendar year. Consequently, most studies in this review, specifically those limited to a single site or with a short duration of data collection were subject to some degree of selection or seasonality bias.

#### Primary outcome

The primary outcome, rate of ED utilization for non-urgent services, across the fifteen studies varied widely. Between 20–25% of the ED visits were classified as ‘avoidable’ or non-urgent” in nature [9,10,35,37]. The studies that limited their samples to children (40% [32], (69%) [33] (68% [31];); and those with cancer (56% [36];) reported higher rates of ED utilizations. Only two studies used multiple measurements of defining ‘non-urgent’ and reported specific estimates for ‘primary care treatable non-urgent visits’ (90.5% - [36]; 27.4% - [5]), while the lowest reported rate was 13.5% when non-urgent was defined based on “ED resource utilization including testing, therapeutics or hospital admission [5].

### Distance decay effects

#### Patient-level pediatric populations

Five studies examined pediatric populations aged 18 and younger in their analysis of distance traveled to the ED [9,30–33]. With the exception of one study in the US [30], the remaining studies were conducted in Western Europe (Belgium, and Germany) and Turkey. All studies except one [31] found empirical evidence indicative of an inverse relationship between distance and ED use of non-urgent services. Specifically, as distance to an ED increased, utilization of non-urgent services declined. Evidence was corroborated using descriptive bivariate analysis in two of these studies [9,30] while the remaining two studies used multivariable analyses controlling for relevant socio-demographic characteristics as covariates [32,33].

#### Patient-level all populations

With the exception of one study [35], five studies examining adults or populations across all ages demonstrated a negative relationship between ED use for non-urgent services and distance traveled [5,10,34,36,37]. Two studies included patients of all ages [10,37]. One study performed a multiple discriminant analysis and determined distance to be a distinguishing factor between patients that sought treatment (stayers) and those that left the ED without seeking any care (leavers) [34]. Four studies reported the distance decay effect based on results from multivariable regression analyses [5,10,36,37]. Of these studies, although the reported magnitudes indicative of a ‘distance decay’ differed, overall, the main findings were consistent with this phenomenon. Three studies demonstrated distance decay using multiple years of data [10,37], or testing for the effect through the use of multiple measurements of ‘non-urgent’ [5], thus making their findings more robust.

#### Ecological studies

Five studies used ecological study designs [9,15,38–40], where data was analyzed on populations or groups rather than individuals. The oldest publication in this category was a study that found evidence in support of distance decay or a negative spatial interaction of distance traveled and ED utilization rates among census tracts in Canada [39]. However, it did not control for any potential confounders. A second descriptive study also reported a decline in ED use with each additional distance band traveled among children less than 16 years of age in Italy across three EDs in three main cities [9]. The remaining three studies in this category reported a moderate evidence of distance decay using large multi-year data [15,38,40]. In these studies, the shortest median distance to a nearest hospital (~3 miles) was reported by McGarry et al. [40] and Okunseri et al. [15], both in the US. Vaz et al. examined distance decay in Portugal and found the most pronounced distance decay effects in the case of medical-surgical EDs providing intermediate level of care when compared to EDs providing basic or complex level of care [38]. While basic EDs are less resourceful, medical-surgical and polyvalent EDs providing intermediate to complex care respectively are staffed with clinicians with greater medical expertise and skills along with the capacity to perform advanced laboratory and imaging examinations.

### Distance decay effects across geographic regions

When differentiated by geographic regions, four studies examining countries in North America using multivariable empirical methods showed a distance decay effect for pediatric, adult or older adult study populations [10,15,37,40]. For countries in the European region, four out of five studies conducting multivariable analysis showed a moderate distance decay effect in Portugal [38], Germany [33], Belgium [32] and France [5]. Finally, a study examining Turkey, a middle-income country, no distance decay was reported for a pediatric study population [31], whereas a small effect was reported among cancer patients visiting the ED for non-urgent care in Korea, a high-income country in Asia [36].

### Potential confounders

With the exception of four studies [9,30,34,39], all others controlled for confounders in their statistical models. Seven studies [5,10,15,32,33,37,38] controlled for access to alternative providers or its proxies by including critical confounders such as location (urban/rural) [15], number of primary health centers or density of medical [5,38] or dental providers [15]; location of alternative providers [10,37], or more specific measures such as patient registered with a general practitioner (GP) [32], or whether the visit took place during out-of-office hours [32] or during PCP office hours [33]. Evidence on the effects of these measures was rather mixed. While Okunseri et al. [15], and Naouri et al. [5] did not report provider density to have any significant effect, physicians’ out-of-office hours were seen to positively associate with ED visits for non-urgent care [5,32]. Two studies that specifically tested for the effect of proximity to Federally Qualified Health Centers (FQHCs), clinics designated for affordable providers in the US, found that as distance to FQHC increased, ED visits for non-urgent care also increased [10,37]. Only one study included transportation mode as a confounder but did not find any significant effect [31].

Among other covariates, seven and five studies respectively found younger age of patients [5,10,31–33,35,37] and/or being a female [5,10,35,37,38] to be positively associated with ED use for non-urgent reasons. The correlation of younger patient age with non-urgent ED use was specifically true for children [31–33]. Among pediatric studies, Pehlivanturk-Kizilkan et al. [36] was the only study that controlled for parental characteristics such as father’s employment status, and age of both parents. While father’s employment status played a pivotal role in reducing pediatric use of EDs for non-urgent care, younger age of parents was associated with lower ED use for non-urgent reasons [31].

Chen et al. tested the moderating effects of types of insurance users on distance [10,37]. Some sub-groups such as those with public insurance in California, US [37], or commercial insurance in South Carolina, US [10] that were living closer to EDs, were seen to be using it more for non-urgent care. Chen et al.. (2015b) also reported African American and/or those with public insurance to be frequent users of EDs for non-urgent care [10]. An additional non-US study reported a significant negative influence of having private supplemental insurance on ED use in France [5].

Additionally, a few studies showed a positive influence of patient’s education level on reducing non-urgent ED use [5,38]. The higher the education, the lower the non-urgent ED use. Also, five studies [5,31,33,36,38] reported an indicator of overall health condition measured by the absence of chronic disease or preexisting conditions [31,33], the type or status of cancer [36], admission to ED in the recent past [31,36], or duration of the presenting problem [5,35] to have a significant effect on ED use for non-urgent care. Lastly, one of the ecological studies controlled for aggregate level population measures such as mortality rate, mean age, homelessness, and unemployment rates to have a significant positive influence on ED use for non-urgent services [38].

### Quality of evidence and risk of bias

In Table 3, the quality of the body of evidence for the presence of distance decay was assessed as moderate, low or very low according to GRADE criteria. Over half (eight) of the studies presenting moderate level of evidence relied on large samples, multi-year patient-level data, and included empirical models controlling for potential confounders [5,10,15,32,36–38,40].

Three studies reported lower evidence [31,33,35]. While Bianco et al. reported a negative distance decay, it failed to achieve statistical significance [35]. Pehlivanturk-Kizilkan, did not report any meaningful findings [31]. Guckert et al. reported a negative association but did not present the magnitude of this relation in the study [33]. Four studies reported very low quality of evidence in support of distance decay as these studies did not control for any potential confounders [9,30,34,39]. The remaining studies provided their results in the form of odds ratios generated from logistic regressions or slope coefficients/elasticities from other regression models [5,10,15,32,36–38,40].

### Appraisal of studies

Table 4 shows the studies that were graded as satisfactory, based on a modified version of a well-established quality appraisal tool recommended by Crombie [42]. The quality of the selected studies was found to be mostly satisfactory. All fifteen papers met five of the fifteen criteria.

To an extent, a subjective element was involved in the assessment of the outcome variable and the classification of non-urgent visits in all studies. These studies relied on pre-determined algorithms whose validity were not proven for the specific study sites. Additionally, a single or group of physicians assessed the urgency of conditions. Nevertheless, the use of algorithms or pre-determined assessments is well grounded in the scholarly literature and widely adopted in the context of ED use. The other limitations of the studies were related to the description of data [15,34,38], consistency in the number of subjects reported [15,33,36], or assessment of statistical significance [33]. None of the studies addressed all potential biases. While some limited their methodology to a univariate analysis [9,30,39], others did not sufficiently describe the empirical strategy [34], or address sample representativeness [5,15,31,32,35,36,38], or controlled for all potential confounders [10,37,40]. Hilker et al. did not compare their findings to previous reports or discuss implications of their results to real-life [30], while one study failed to discuss their null findings with respect to ED use [35].

## Discussion

We identified fifteen observational studies for this systematic review, of which eight provided moderate evidence of a negative relationship between distance to an ED and utilization of non-urgent services across all populations, including children and adults, as well as at the ecological level. Statistically significant distance decay effects were demonstrated based on samples ranging from small to large drawn from surveys administered to patients as well as administrative databases linked to EDs of single or multiple hospitals in single states or larger geographic regions.

Majority of the studies (eleven of fifteen) included in this review examined the distance decay effect using a multivariable regression model controlling for potential confounders [5,10,15,31–33,35–38,40]. Of these eleven, eight of them reported moderate evidence of a distance decay effect in the utilization of ED services for non-urgent care indicating that individuals further away from an ED were less likely to use it for non-urgent care [5,10,15,32,36–38,40]. These eight studies were conducted in the United States, Canada, and countries in the European Union and Korea. Although the magnitude of distance decay differed, the effect was consistently present across different populations including pediatric, geriatric or patients of all ages. Additionally, studies examining the moderating role of having (or not having) insurance also reported consistent findings on the presence of a distance decay effect. Specifically, it was evident that populations with public insurance demonstrated a larger distance decay effect compared to counterparts on private insurance [37]. This finding implies that ED utilization rate declined more steeply with every additional unit of distance traveled for populations on public insurance, when compared to counterparts on private insurance. In contrast, one study specific to cancer patients in South Korea reported a smaller distance decay effect indicating that longer distances do not deter individuals with a medical history such as cancer from seeking potentially avoidable ED care [36].

Several factors were discussed to interpret distance decay effects associated with the utilization of ED services for non-urgent care across the fifteen studies. The primary explanation offered in the prior literature is in terms of travel costs, both direct costs and time [12]. A higher travel cost of using an ED located further away from where one lives can deter utilization, specifically when the nature of the services sought is not perceived as urgent or life-threatening [12]. Additionally, multiple studies in our review argued that having an ED close to one’s place of residence was one of the factors driving use, especially for non-urgent conditions. Two pediatric studies reported locational convenience as a common factor to explain the distance decay phenomenon [32,33]. Similarly, findings on adult populations reported in two studies indicated convenience of using a nearby ED as a factor driving non-urgent use [5,36]. Furthermore, while Hilker did not empirically test for a distance decay effect but instead argued that the presence of expressways in the US made it much more convenient for parents living in outlying areas to access hospitals with more ease than areas not served by expressways [30], thus implying travel time might be an indicator of convenience.

An alternative explanation forwarded in two studies in our review relates to why parents may choose to take their children to an ED due to a possible lack of access to primary care providers or first line providers in closer proximity [32,33]. Guckert et al. also argued that parents are more likely to perceive pediatric EDs to be better equipped with resources and staff to diagnose their child’s condition in the quickest possible manner, thereby relieving their own anxiety [33]. Mistaken perceptions of clinical urgency combined with the need to relieve one’s anxiety were discussed for adult patients too [5,36] but might have played a greater role among pediatric populations than among adults.

Another factor used to explain distance decay in non-urgent ED use relates to availability or access to primary care providers in the community. Several studies in the prior literature showed a positive association between ED use and physician practice characteristics, such as practice hours or distance to clinics, although these studies did not examine effects of distance on ED utilization for non-urgent services [43–45]. Despite this prior evidence, not all studies in our review accounted for provider characteristics in their analyses. Only two of the fifteen studies examined the effect of proximity to alternative providers and, as expected, reported a positive relationship between distance to community health centers and ED utilization for non-urgent services [10,37].

For non-urgent services, ED use declines when distance of a patient’s residence to the nearest primary care provider decreases. Consistently, another study found a significant relationship between provider density and ED use in the context of Portugal [38]. Additionally, three studies suggested that adult patients’ tended to use EDs as a surrogate for family physicians outside of office hours [10,37,38]. Similarly, studies that included whether an ED visit took place during out-of-office physicians’ hours demonstrated the adverse effect of non-availability of community providers on ED use [5,32]. Thus, these studies linked findings on distance decay in non-urgent ED use to potential barriers to care in the community.

With regard to non-urgent utilization rates, our outcome variable, a wide variability was reported that might have been due to a range of factors, such as variation across study samples, location of hospitals, or catchment areas hospitals served. Additionally, differences in reported rates might have stemmed from the way ‘non-urgent care’ was defined, whereas country-specific structuring of health care systems to meet medical needs may have also been a contributing factor [46]. Among the nine countries discussed in this review, the United States is the only country that does not offer universal health coverage, while others have a socialized health care system with most services covered by a public insurance scheme in Canada, France, Germany, Belgium, Italy, Turkey, Portugal and Korea [47]. Moreover, EDs in all of these countries, including the United States, cater to all types of patients irrespective of the level of medical urgency [48]. Given these factors, policies and interventions are needed to reduce non-urgent use of the EDs for conditions that could be treated in primary care settings [49].

In summary, patient-perceptions (of convenience or anxiety) and access-related factors discussed in the studies we reviewed indicate the potential of three broad set of policy recommendations to reduce unnecessary use of ED care for non-urgent reasons. Re-structuring benefits and generosity of plans offered under publicly insured schemes may help to increase access in areas with a lower supply of physicians. Among the studies that reported non-urgent ED use, about one in four ED visits were considered inappropriate [37], while people with public insurance, such as the state-funded Medicaid program (in the US) or uninsured, were more likely to use the ED for non-urgent care [10]. A French study reported similar findings showing that patients without supplemental health insurance, typically used by people to pay for co-payments or services not covered by public insurance schemes, were more likely to use EDs for non-urgent care [5].

Second, evidence of distance decay in countries with mandatory public health insurance [31–33] indicates that reducing out of pocket costs by itself may not address the problem of non-urgent ED use. Alternative efforts toward improving access to primary or first-line care along with patient education and awareness should thus be considered. Three of the pediatric studies reported a non-urgent utilization rate of 40% or over in countries with socialized health care systems [31–33]. In fact, in Germany, PCP and ED care are both free of any charge. Yet, a disproportionate share of the ED visits were classified as non-urgent among children [33]. Such high rates of ED utilization call for efforts to improve access to primary care by setting up facilities offering triage after-hour calls at physicians’ offices such as in the United States where it has shown to be associated with patient satisfaction, safety and cost savings [50]. Provision of such options would help in alleviating the anxiety of especially vulnerable populations including parents of infants and young children, those with compromised immune systems such as cancer patients as seen in Oh et al. [36], or older adults. Lastly, a longer-term option would be to increase the overall supply or volume of practicing physicians per capita, either through the setting up of more medical schools or by making cross-border flows of physicians less restrictive [51].

### Strengths, limitations and future work

This systematic review was guided by a protocol developed following the PRISMA methodology. To the best of our knowledge, this is the first review on the topic of distance decay that provides a narrative synthesis of findings at the individual and population levels, presenting effects for different sub-groups of populations and covering different countries. However, it is subject to some limitations. First, the eligibility of articles was assessed by two reviewers, and the process was completed based on authors’ subjective judgment. The authors extensively discussed discrepancies related to studies in order to reach a consensus. Second, although articles were searched extensively across five databases, our search may have still missed some studies reporting on this topic. Additionally, non-English publications and conference abstracts were excluded.

Based on GRADE, eight out of the fifteen studies presented moderate level of evidence in support of distance decay [5,10,15,32,36–38,40]. However, several studies did not adjust for all potentially confounding variables [9,10,15,30,32,34–37,39]. Therefore, future empirical studies are needed that interpret the distance decay phenomenon after controlling for potential confounders including patient-, household and provider characteristics (such as, location or office hours, parents’ education, etc.). Additionally, only two studies by Wartman et al. [34] and Naouri et al. [5] used a mixed-methods approach and interviewed their study participants for a deeper understanding of the reasons and motivations for seeking non-urgent ED care. Along similar lines, future studies are needed to triangulate empirical findings with qualitative data that could be obtained by surveying families or patients living at different distances from EDs. Another area worthy of future investigation is using a more precise measurements of distance, such as road or transportation networks or travel time.

## Conclusion

Applying the PRISMA methodology, fifteen studies investigating the association between distance and non-urgent visits to emergency departments were selected and reviewed. Of these fifteen studies, eight measured and found moderate evidence of a distance decay effect, indicating a decrease in ED use for non-urgent care with each additional unit of distance traveled. The remaining seven studies presented low or very low evidence of a negative relationship between distance traveled and ED use. This systematic review recommends policy-makers ways to reduce non-urgent use of the EDs through re-structuring public benefits offered under health care systems and improving accessibility to providers in the community along with patient education and awareness. Widening the availability of physicians in the community might additionally help in relieving some of the pressure on EDs to cater to non-urgent health needs of populations, especially those bypassing their community-based providers due to non-availability of appointments.

## References

[1] Trzeciak S, Rivers EP. Emergency department overcrowding in the United States: an emerging threat to patient safety and public health. Emerg med J. 2003;20:402–19. Epub 2003/09/05. doi: 10.1136/emj.20.5.402 12954674 PMC1726173

[2] Young GP, Wagner MB, Kellermann AL, Ellis J, Bouley D. Ambulatory visits to hospital emergency departments: patterns and reasons for use. JAMA. 1996;276:460–465. doi: 10.1001/jama.1996.03540060036032 8691553

[3] Lee JE, Sung JH, Ward WB, Fos PJ, Lee WJ, Kim JC. Utilization of the emergency room: impact of geographic distance. Geospat Health. 2007;1:243–253. doi: 10.4081/gh.2007.272 18686249

[4] Herndon JB, Chaney M, Carden D. Health literacy and emergency department outcomes: a systematic review. Ann Emerg Med. 2011;57:334–345. doi: 10.1016/j.annemergmed.2010.08.035 21035902

[5] Naouri D, Ranchon G, Vuagnat A, Schmidt J, El Khoury C, Yordanov Y. Factors associated with inappropriate use of emergency departments: findings from a cross-sectional national study in France. BMJ Qual Saf. 2020;29:449–464. doi: 10.1136/bmjqs-2019-009396 PMC7323738 31666304

[6] Hooker EA, Mallow PJ, Oglesby MM. Characteristics and trends of emergency department visits in the United States (2010 – 2014). J Emerg Med. 2019;56:344–351. doi: 10.1016/j.jemermed.2018.12.025 30704822

[7] Ludwick A, Fu R, Warden C, Lowe RA. Distances to emergency department and to primary care provider’s office affect emergency department use in children. Acad Emerg Med. 2009;16:411–417. doi: 10.1111/j.1553-2712.2009.00395.x 19388919

[8] Williams A, O’Rourke P, Keogh S. Making choices: why parents present to the emergency department for non-urgent care. Arch Dischildhood. 2009;94:817. doi: 10.1136/adc.2008.149823 19395399

[9] Valent F, Busolin A. Distance to the pediatric emergency department and nonurgent visits in friuli venezia giulia, Italy. Pediatr Emer Care. 2018;34:193–197. doi: 10.1097/PEC.0000000000000852 27749627

[10] Chen BK, Cheng X, Bennett K, Hibbert J. Travel distances, socioeconomic characteristics, and health disparities in nonurgent and frequent use of hospital emergency departments in south carolina: a population-based observational study. BMC Health Serv Res. 2015;15:203. doi: 10.1186/s12913-015-0864-6 25982735 PMC4448557

[11] Andersen R, Newman JF. Societal and individual determinants of medical care utilization in the United States. The Milbank Memorial Fund Quartely: Health And Society. 1973;51:95–124. doi: 10.2307/3349613 4198894

[12] Demir I, Khan M. Estimating the effects of travel distance and costs on Emergency Department (ED) utilization: learnings from individual level data. Int J Econ Finance Stud. 2017;9:64–76.

[13] Bergeron P, Courteau J, Vanasse A. Proximity and emergency department use: multilevel analysis using administrative data from patients with cardiovascular risk factors. Can Fam Physician. 2015;61:e391–e7. 26505061 PMC4541449

[14] Stock R. Distance and the utilization of health facilities in rural Nigeria. Soc Sci Med. 1983;17:563–570. doi: 10.1016/0277-9536(83)90298-8 6879255

[15] Okunseri C, Vanevenhoven R, Chelius T, Beyer KMM, Okunseri E, Lobb WK, et al. Travel distances by wisconsin medicaid enrollees who visit emergency departments for dental care. J Public Health Dent. 2016;76:213–219. doi: 10.1111/jphd.12138 26797756 PMC4956604

[16] Sun Y-Y, Lin P-C. How far will we travel? A global distance pattern of international travel from both demand and supply perspectives. Tourism Econ. 2019;25:1200–1223. doi: 10.1177/1354816618825216

[17] Asplin BR, Magid DJ, Rhodes KV, Solberg LI, Lurie N, Camargo CA Jr. A conceptual model of emergency department crowding. Ann Emerg Med. 2003;42:173–180. Epub 2003/07/29. doi: 10.1067/mem.2003.302 12883504

[18] Hoot NR, Aronsky D. Systematic review of emergency department crowding: causes, effects, and solutions. Ann Emerg Med. 2008;52:126–136. Epub 2008/04/25. doi: 10.1016/j.annemergmed.2008.03.014 18433933 PMC7340358

[19] Naavaal S, Kelekar U. Opioid prescriptions in emergency departments: findings from the 2016 national hospital ambulatory medical care survey. Preventive med. 2020;136:106035. doi: 10.1016/j.ypmed.2020.106035 32112795

[20] Kelekar U, Naavaal S. Dental visits and associated emergency department - charges in the United States: nationwide emergency department sample, 2014. J Am Dent Assoc. 2019;150:305–12.e1. doi: 10.1016/j.adaj.2018.11.021 30922460

[21] Althaus F, Paroz S, Hugli O, Ghali WA, Daeppen J-B, Peytremann-Bridevaux I, et al. Effectiveness of interventions targeting frequent users of emergency departments: a systematic review. Ann Emerg Med. 2011;58:41–52.e42. doi: 10.1016/j.annemergmed.2011.03.007 21689565

[22] Morgan SR, Chang AM, Alqatari M, Pines JM. Non-emergency department interventions to reduce ED utilization: a systematic review. Acad Emerg Med. 2013;20:969–985. Epub 2013/10/17. doi: 10.1111/acem.12219 24127700 PMC4038086

[23] Carr BG, Addyson DK. Geographic information systems and emergency care planning. Acad Emerg Med. 2010;17:1274–1278. doi: 10.1111/j.1553-2712.2010.00947.x 21416801

[24] Carret MLV, Fassa ACG, Domingues MR. Inappropriate use of emergency services: a systematic review of prevalence and associated factors. Cad Saúde Pública. 2009;25:7–28. doi: 10.1590/S0102-311X2009000100002 19180283

[25] Uscher-Pines L, Pines J, Kellermann A, Gillen E, Mehrotra A. Emergency department visits for nonurgent conditions: systematic literature review. Am J Manag Care. 2013;19:47–59. 23379744 PMC4156292

[26] Huang B, Kelekar U. Distances to emergency departments and non-urgent utilization of medical care: a systematic review. Prospero 2023 Crd42023398674. 2023. Available from: https://www.crd.york.ac.uk/prospero/display_record.php?ID=CRD42023398674 10.1080/16549716.2024.2353994 PMC11149577 38828477

[27] Page MJ, McKenzie JE, Bossuyt PM, Boutron I, Hoffmann TC, Mulrow CD, et al. The PRISMA 2020 statement: an updated guideline for reporting systematic reviews. BMJ. 2021;372:n71. doi: 10.1136/bmj.n71 33782057 PMC8005924

[28] Higgins J, Thomas J, Chandler J, Cumpston M, Li T, Page M, et al. Cochrane handbook for systematic reviews of interventions version 6.3 (updated February 2022). Cochrane. 2022. Available from: https://training.cochrane.org/handbook

[29] Covidence systematic review software. Melbourne, Australia: Veritas Health Innovation. Available from: http://www.covidence.org

[30] Hilker TL. Nonemergency visits to a pediatric emergency department. JACEP. 1978;7:3–8. doi: 10.1016/S0361-1124(78)80248-2 619171

[31] Pehlivanturk-Kizilkan M, Ozsezen B, Batu ED. Factors affecting nonurgent pediatric emergency department visits and parental emergency overestimation. Pediatr Emer Care. 2022;38:264–268. doi: 10.1097/PEC.0000000000002723 35507379

[32] Benahmed N, Laokri S, Zhang WH, Verhaeghe N, Trybou J, Cohen L, et al. Determinants of nonurgent use of the emergency department for pediatric patients in 12 hospitals in Belgium. Eur J Pediatr. 2012;171:1829–1837. doi: 10.1007/s00431-012-1853-y 23064744

[33] Guckert L, Reutter H, Saleh N, Ganschow R, Muller A, Ebach F. Nonurgent visits to the pediatric emergency department before and during the first peak of the COVID-19 pandemic. Int J Pediatr. 2022;2022:7580546. doi: 10.1155/2022/7580546 35242194 PMC8886764

[34] Wartman SA, Taggart MP, Palm E. Emergency room leavers: a demographic and interview profile. J Community Health. 1984;9:261–268. doi: 10.1007/BF01338726 6434597

[35] Bianco A, Pileggi C, Angelillo IF. Non-urgent visits to a hospital emergency department in Italy. Public Health. 2003;117:250–255. doi: 10.1016/S0033-3506(03)00069-6 12966745

[36] Oh TK, Jo YH, Choi JW. Associated factors and costs of avoidable visits to the emergency department among cancer patients: 1-year experience in a tertiary care hospital in South Korea. Support Cancer Ther. 2018;26:3671–3679. doi: 10.1007/s00520-018-4195-0 29740693

[37] Chen BK, Hibbert J, Cheng X, Bennett K. Travel distance and sociodemographic correlates of potentially avoidable emergency department visits in California, 2006–2010: an observational study. Int J Equity Health. 2015;14:30. doi: 10.1186/s12939-015-0158-y 25889646 PMC4391132

[38] Vaz S, Ramos P, Santana P. Distance effects on the accessibility to emergency departments in Portugal. Saude e Sociedade. 2014;23:1154–1161. doi: 10.1590/S0104-12902014000400003

[39] Ingram DR, Clarke DR, Murdie RA. Distance and the decision to visit an emergency department. Social Science & Medicine Part D: Medical Geography. 1978;12:55–62. doi: 10.1016/0160-8002(78)90007-2 644349

[40] McGarry BE, Mao Y, Nelson DL, Temkin-Greener H. Hospital proximity and emergency department use among assisted living residents. J Am Med Dir Assoc. 2023;2023:1349–1355.e5. doi: 10.1016/j.jamda.2023.05.002 PMC10524627 37301223

[41] Balshem H, Helfand M, Schünemann HJ, Oxman AD, Kunz R, Brozek J, et al. GRADE guidelines: 3. Rating the quality of evidence. J Clinical Epidemiol. 2011;64:401–406. doi: 10.1016/j.jclinepi.2010.07.015 21208779

[42] Crombie IK. Pocket guide to critical appraisal. London: BMJ Publishing Group; 1996.

[43] Turnbull J, Martin D, Lattimer V, Pope C, Culliford D. Does distance matter? Geographical variation in GP out-of-hours service use: an observational study. Br J Gen Pract. 2008;58:471–477. doi: 10.3399/bjgp08X319431 18611312 PMC2441507

[44] Wolfson JA, Schrager SM, Khanna R, Coates TD, Kipke MD. Sickle cell disease in California: sociodemographic predictors of emergency department utilization. Pediatr Blood Cancer. 2012;58:66–73. doi: 10.1002/pbc.22979 21360655 PMC3272000

[45] Fishman J, McLafferty S, Galanter W. Does spatial access to primary care affect emergency department utilization for nonemergent conditions? Health Serv Res. 2018;53:489–508. doi: 10.1111/1475-6773.12617 27859257 PMC5785320

[46] Keizer Beache S, Guell C. Non-urgent accident and emergency department use as a socially shared custom: a qualitative study. Emerg med J. 2016;33:47–51. doi: 10.1136/emermed-2014-204039 25841166 PMC4717374

[47] Böhm K, Schmid A, Götze R, Landwehr C, Rothgang H. Five types of OECD healthcare systems: empirical results of a deductive classification. Health Policy. 2013;113:258–269. doi: 10.1016/j.healthpol.2013.09.003 24095274

[48] Zibulewsky J. The emergency medical treatment and active labor act (EMTALA): what it is and what it means for physicians. Proc (Bayl Univ Med Cent). 2001;14:339–346. doi: 10.1080/08998280.2001.11927785 16369643 PMC1305897

[49] Van den Heede K, Van de Voorde C. Interventions to reduce emergency department utilisation: a review of reviews. Health Policy. 2016;120:1337–1349. doi: 10.1016/j.healthpol.2016.10.002 27855964

[50] Bunik M, Glazner JE, Chandramouli V, Emsermann CB, Hegarty T, Kempe A. Pediatric telephone call centers: how do they affect health care use and costs? Pediatrics. 2007;119:e305–13. doi: 10.1542/peds.2006-1511 17272593

[51] Wiedermann CJ. Revitalizing general practice: the critical role of medical schools in addressing the primary care physician shortage. Healthcare (Basel). 2023;11:1820. doi:10.3390/healthcare11131820 37444654 PMC10340705

